# Smoking and Diabetes: Is the Association Mediated by Adiponectin, Leptin, or C-reactive Protein?

**DOI:** 10.2188/jea.JE20140055

**Published:** 2015-02-05

**Authors:** Esayas Haregot Hilawe, Hiroshi Yatsuya, Yuanying Li, Mayu Uemura, Chaochen Wang, Chifa Chiang, Hideaki Toyoshima, Koji Tamakoshi, Yan Zhang, Nobuo Kawazoe, Atsuko Aoyama

**Affiliations:** 1 Department of Public Health and Health Systems, Nagoya University Graduate School of Medicine, Nagoya, Japan; 1 名古屋大学大学院医学系研究科国際保健医療学・公衆衛生学; 2 Department of Public Health, School of Medicine, Mekelle University, Mekelle, Ethiopia; 2 エチオピア メケレ大学 公衆衛生学; 3 Department of Public Health, Fujita Health University School of Medicine, Toyoake, Aichi, Japan; 3 藤田保健衛生大学医学部公衆衛生学; 4 Anjo Kosei Hospital, Anjo, Aichi, Japan; 4 安城更生病院; 5 Department of Nursing, Nagoya University Graduate School of Medicine, Nagoya, Japan; 5 名古屋大学大学院医学系研究科看護学専攻

**Keywords:** adiponectin, C-reactive protein, leptin, mediation analysis, smoking, type 2 diabetes mellitus

## Abstract

**Background:**

Although the association between cigarette smoking and risk of type 2 diabetes is well established, its mechanisms are yet to be clarified. This study examined the possible mediating effects of adiponectin, leptin, and C-reactive protein (CRP) concentrations on the smoking-diabetes association.

**Methods:**

Between 2002 and 2011, we followed 3338 Japanese workers, aged 35–66 years, who were enrolled in the second Aichi workers’ cohort study. We used multivariable-adjusted Cox regression models to determine the hazard ratios and respective 95% confidence intervals (CIs) of the association between smoking status and risk of diabetes. A multiple mediation model with bootstrapping was used to estimate the magnitude and the respective bias-corrected (BC) 95% CIs of the indirect effects of smoking on diabetes through the three biomarkers.

**Results:**

Relative to never smokers, the risk of diabetes was significantly elevated in current (hazard ratio 1.75, 95% CI 1.25–2.46) and ex-smokers (hazard ratio 1.54, 95% CI 1.07–2.22). The indirect effects of smoking on diabetes through adiponectin levels were statistically significant among light (point estimate 0.033, BC 95% CI 0.005–0.082), moderate (point estimate 0.044, BC 95% CI 0.010–0.094), and heavy smokers (point estimate 0.054, BC 95% CI 0.013–0.113). In contrast, neither the indirect effects of smoking on diabetes through leptin nor CRP levels were significant, as the corresponding BC 95% CIs included zero.

**Conclusions:**

In our analysis, adiponectin concentration appeared to partially mediate the effect of smoking on diabetes, while leptin and CRP levels did not.

## Abstract (ja)

**背景：:**

喫煙が2型糖尿病発症リスクに関連することはよく知られているが、そのメカニズムは十分解明されていない。そこで、その関連がアディポネクチン、レプチン、C反応タンパク（CRP）によって説明されるか検討した。

**方法：:**

愛知職域コホート（第2次）に参加した35-66歳の男性従業者3,338名を2002年から2011年まで追跡した。多変量調整Cox回帰モデルを用いてベースライン時喫煙状況の糖尿病発症に対するハザード比と95％信頼区間を計算した。喫煙が上記3つのバイオマーカーを介して糖尿病発症リスクに与える間接的な「影響」の大きさは多変量仲介モデルを用いて推定し、そのバイアス調整95%信頼区間（BC 95%CI）はブートストラップ法によって求めた。

**結果：:**

非喫煙者に比し、現喫煙者の糖尿病発症リスクは有意に高かった（ハザード比：1.75、95%CI：1.25-2.46）。喫煙の糖尿病発症に対するアディポネクチンを介する「影響」の大きさの推定値（BC 95%CI）は、軽度、中等度、重度の喫煙でそれぞれ0.033（0.005-0.082）、0.044（0.010-0.094）、0.054（0.013-0.113）であった。レプチンやCRPを介する影響は統計学的に有意ではなかった。

**結論：:**

喫煙の糖尿病発症に対する「影響」の一部はアディポネクチンによって仲介されていると考えられた。

## INTRODUCTION

Cigarette smoking is independently associated with incidence of type 2 diabetes mellitus (DM).^1^^,^^2^ Although details behind the mechanism of this association are not yet fully understood, promotion of central obesity, hypercortisolaemia, nicotine-induced impaired beta-cell function, and elevations in inflammatory markers and oxidative stress caused by smoking are suspected.^2^^,^^3^

Adiponectin and leptin are the most abundant adipocytokines produced by adipocytes and have anti- and pro-inflammatory properties, respectively.^4^^,^^5^ Smoking may cause a decrease in adiponectin levels,^6^^–^^10^ and decreased adiponectin levels have been consistently associated with DM incidence.^11^^–^^14^ With regard to leptin, previous studies on its association with smoking have often yielded conflicting results: reports of decrease,^15^^–^^18^ no effect,^19^ or increase^20^^,^^21^ in the leptin levels of smokers are all available. Similarly, reports on the association between serum leptin levels and DM incidence have been inconsistent: significant associations have been documented in some^22^^–^^24^ but not all^25^^–^^27^ studies.

C-reactive protein (CRP), an acute-phase reactant produced primarily in the liver, has been shown to be a sensitive, systemic biomarker of inflammation.^28^ Several studies have consistently reported a positive association between smoking and CRP levels.^29^^–^^33^ A number of prospective studies have also described the association between circulating CRP levels and DM incidence, with some demonstrating an independently positive association,^34^^–^^36^ while others show no association.^25^^,^^26^^,^^37^^,^^38^

We hypothesized that serum levels of adiponectin, leptin, and CRP could potentially mediate the association between smoking and incidence of DM. We focused on these three biomarkers because they are the most common biomarkers reported in the literature in relation to smoking and DM. We evaluated our hypothesis using data obtained from the second Aichi workers’ cohort study.^39^

## METHODS

### Study population

The second Aichi workers’ cohort study is an ongoing study on cardiovascular diseases among civil servants aged 35 to 66 years in Aichi Prefecture, located in central Japan. Baseline information was collected from 6648 individuals in 2002 through self-administered questionnaires and mandatory annual health check-ups provided by the worksites of study subjects. We excluded subjects with missing values for the following variables: smoking status (*n* = 100); adiponectin, CRP, or leptin levels (*n* = 2613); and other covariates (*n* = 332). Prevalent cases of DM (*n* = 265), diagnosed by self-reported medication use or baseline fasting glucose level ≥126 mg/dL, were also excluded, leaving 3338 subjects for the present analysis. The excluded subjects were not significantly different from those included in the analyses with respect to distribution of variables, including sex, age, smoking status, and DM incidence.

Subjects were followed until they retired. Workers who were reemployed after their retirement age of 60 years were kept in the cohort until they re-retired. Those who retired and were not reemployed were contacted by mail. However, those who did not provide their mailing address were censored at the time of retirement. There were no significant differences between retired subjects who provided mailing addresses and those who did not with regard to their smoking status, body mass index (BMI), and DM incidence. The study protocol was approved by the Ethics Review Committee of Nagoya University School of Medicine, Nagoya, Japan.

### Measurement of exposures, confounders, and outcomes

#### Smoking metrics at baseline

Information on smoking status was acquired through a self-administered questionnaire. Subjects initially responded to an item that classified them as never, ex-, or current smokers. Ex-smokers were defined as those who do not currently smoke cigarettes but had previously smoked for at least a year. Both current and ex-smokers were asked to report the average number of cigarettes they smoke or had smoked per day and the age at which they started smoking. Ex-smokers were also asked to specify the age at which they quit smoking. Duration of smoking for ex-smokers at baseline was calculated in years by subtracting age at the time of cessation from the baseline age. If they had quit more than one time, the subjects were asked to specify the longest duration for which they had abstained from smoking. We categorized current smokers as light (1–19 cigarettes per day), moderate (20–29 cigarettes per day), and heavy smokers (≥30 cigarettes per day). Assuming 20 cigarettes per pack, pack-years of smoking were estimated using the formula, “(cigarettes per day/20) × years smoked”.

#### Adiponectin, leptin, and CRP

Venous blood samples were drawn from each subject after at least 8 h of fasting. Serum samples were stored at −80°C until biochemical assay. Adiponectin concentration was determined in a commercial laboratory using an enzyme-linked immunosorbent assay (Otsuka Pharmaceutical Co., Ltd., Tokyo, Japan), for which the laboratory reports intra-assay coefficients of variation of 6.0% to 8.6%. Leptin concentrations were measured via radioimmunoassay (Human Leptin RIA Kit; Linco Research, Inc., St. Charles, MO, USA) in a commercial laboratory. The detection limit of the leptin assay was 0.5 ng/mL, and the inter-assay coefficients of variation were 1.79% and 1.75% for low- and high-concentration controls, respectively. High-sensitivity CRP was measured by latex nephelometry (BNII; Siemens AG, Erlangen, Germany). The assay was sensitive enough to detect 0.02 mg/L of CRP with an inter-assay coefficient of variation of <4.0%.

#### Fasting blood glucose and other laboratory measurements

Fasting blood glucose was enzymatically determined via the hexokinase method. Insulin concentration was measured by solid-phase radioimmunoassay (RIABEAD II; Dinabot Co., Ltd., Chiba, Japan). Insulin resistance was evaluated with a homeostasis model assessment (HOMA2-IR) using the University of Oxford Diabetes Trials Unit’s HOMA Calculator software (downloaded at http://www.dtu.ox.ac.uk). Total cholesterol and triglycerides were also measured via enzymatic methods, while high-density lipoprotein cholesterol (HDL-C) was determined via the phosphotungstate method.

#### Other Covariates

Dietary habits during the preceding month were assessed using a validated self-administered brief diet history questionnaire. Total energy and nutrient intakes were estimated using an ad hoc computer algorithm developed for nutrient calculation of the brief diet history questionnaire, with reference to the standard tables of food composition in Japan.^40^ Alcohol consumption was calculated by multiplying weekly frequency and the amount drunk on each occasion, with values then converted into grams of ethanol per day. Physically active individuals were defined as those who self-reported to be engaged in a moderate or vigorous leisure-time exercise for a total of 60 minutes or more for more than a day per month. We also assessed self-reported family history of DM among the subjects’ first-degree relatives.

#### Ascertainment of incident diabetes

Incident cases of DM were ascertained using two sources of data: annual mandatory health checkups at work places and questionnaire surveys. As the health checkups were usually carried out from September to December, we defined the date of DM onset as July 1 of the year when fasting glucose level first went beyond 126 mg/dL. The self-administered questionnaire surveys were conducted between 2004 and 2011, in which participants reported their detailed medical histories of various conditions, including DM. Whenever appropriate, participants revealed the year of DM diagnosis as well as the name and address of their present or past physician. Written consent for our access to the participants’ medical records from their physicians was also obtained. For cases with consent, the accuracy of self-reports (95%) was previously confirmed by reviewing their medical records, and the details of the validation study have been reported elsewhere.^39^

### Statistical analyses

First, we generated descriptive statistics to determine whether there were differences in covariates among the five smoking categories (never, ex-, light, moderate, and heavy smokers). Variables with a skewed distribution (i.e., consumptions of alcohol and sugar, triglyceride, insulin, HOMA2-IR, adiponectin, leptin, and CRP levels) were log-transformed prior to further analyses to approximately normalize their distributions, and they were expressed as geometric means and 95% confidence intervals (CIs). Other continuous variables were expressed using means and standard deviations, while percentages were used for categorical variables. Analysis of variance (ANOVA) or χ^2^ tests were used, as appropriate, to compare subjects’ characteristics among the five smoking categories.

We used Cox proportional hazards regression models to estimate hazard ratios (HRs) and respective 95% CIs of the risk of DM among ex-smokers and the 3 categories of current smokers compared to never smokers. The models were adjusted for potential baseline confounders, including continuous variables of BMI, mean arterial pressure, sleep duration, total energy intake, alcohol consumption, sugar consumption, total cholesterol to HDL-C ratio, triglycerides, adiponectin, leptin, CRP, HOMA2-IR, as well as dichotomous variables of physical activity (yes/no) and family history of DM (yes/no). A stepwise backward elimination procedure was used to exclude from the final model those variables that did not substantially affect the results (*P* > 0.1). Age (continuous) and sex (dichotomous) were forced into the model at all times. The proportionality assumption was verified with log-log plots and by the use of Shoenfeld residuals. There was a tendency toward non-proportionality for the association between smoking status and incidence of DM. However, sensitivity analysis by logistic regression showed estimates similar to the Cox models, indicating no large violation of the proportionality assumption (results not shown).

**Figure 1.**
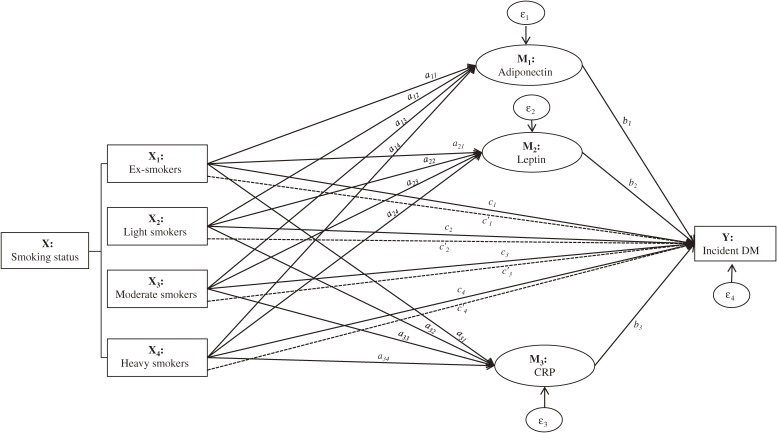
A multiple mediation model illustrating potential mediatory roles of adiponectin, leptin, and CRP concentrations in the smoking-diabetes association. This figure demonstrates how we estimated the indirect effects through the proposed mediators of being in one smoking category (X_1_, X_2_, X_3_, or X_4_) on DM incidence (Y) relative to the never smokers category. The ‘c’ paths represent the overall association—relative total effect—of being in a smoking category with DM incidence, and are equivalent to the unstandardized regression coefficients (β). Mediation is said to occur when (part of) this overall association is explained by a hypothesized mediator variable: adiponectin (M_1_), leptin (M_2_), and CRP (M_3_). Associations of the potential mediators with each smoking category (*a*-paths) and their associations with DM incidence independent *of* one another and of the smoking category (paths *b_1_*, *b_2_*, and *b_3_*) are assessed simultaneously. The product of regression coefficients *a*’s and *b*’s (*ab*’s) quantifies the relative indirect effect of being in one smoking category, relative to the reference, on DM incidence through the mediators. For instance, the relative indirect effect of being an ex-smoker (X_1_) relative to never smokers on DM incidence via adiponectin (M_1_), leptin (M_2_), and CRP (M_3_) is given by products *a_11_b_1_*, *a_21_b_2_*, and *a_31_b_3_*, respectively. The part of the relative total effect (*c_1_*) not explained by these mediating paths is the relative direct effect (*c*′*_1_*) of being an ex-smoker on DM incidence. Similarly, the relative total direct and indirect effects of being in the other smoking categories on DM incidence relative to the never smokers can be represented by the respective paths linking the independent, dependent, and potential mediator variables. DM, diabetes mellitus; CRP, C-reactive protein.

Second, we conducted multiple mediation analysis using the PROCESS procedure for SPSS.^41^ Standard path-analytic approaches^41^^–^^43^ were followed to assess: 1) the effect of being an ex-(X_1_), light (X_2_), moderate (X_3_), or heavy (X_4_) smoker on DM incidence (Y) relative to never smokers (reference group)—*c* paths; 2) the differences between each smoking category and the reference group on the level of the proposed mediators (Ms), adiponectin (M_1_), leptin (M_2_), and CRP (M_3_)—*a* paths; and 3) the association between the Ms and Y while statistically equating the groups on average on X and the other potential mediators in the model—*b* paths (Figure 1). Coefficients for *a* paths were estimated using ordinary least-squares regression, whereas logistic regression was used for the coefficients of the *b* and *c* paths. All of these analyses were adjusted for the covariates included in the Cox model mentioned above. Unstandardized coefficients (β) with their standard errors (SE) are reported for each model. The *c* paths represent the total effects of Xs on Y relative to the reference group unadjusted for the group differences in Ms. These can be apportioned into the direct effects of being in one X group on Y relative to the reference group adjusted for the group differences in Ms (*c*′ paths) and the indirect effects through the Ms of being in one X group relative to the reference group on Y (estimated by the products of *a* and *b* paths—*ab*’s). Bias-corrected (BC) 95% CIs for the indirect effects were generated from 10 000 bootstrap samples, and statistical significance is indicated when the CI values do not cross zero. Bootstrapping is recommended for testing of indirect effects because it does not assume normality in sampling distribution.^43^

All statistical analyses were conducted using IBM SPSS Statistics for Windows software, Version 21.0 (IBM Corp, Armonk, NY, USA), and all tests were two-sided with the significance level set at *P* < 0.05.

## RESULTS

### Baseline characteristics

Characteristics of the study population across the five categories of smoking status at baseline are shown in Tables 1 and 2. Compared to never smokers, heavy smokers were characterized by lower serum levels of adipocytokines (adiponectin and leptin), higher levels of CRP, and more adverse cardio-metabolic risk factors. Incident diabetic cases were also more likely to be in the heavy smoker category.

**Table 1.** Demographic and lifestyle characteristics of study subjects at baseline by smoking status, Aichi, 2002–2011

Characteristics	Smoking status at recruitment					*P*-value^b^

	Never smokers	Ex-smokers	Current smokers, by cigarettes per day			

			Light smokers (<20)	Moderate smokers (20–<30)	Heavy smokers (30+)	
*n*	1608	776	264	421	269	
Demographic factors						
Sex, % men	58.0	94.5	84.1	96.9	99.3	<0.001
Age, years	46.2 (7.1)	49.7 (6.5)	47.6 (7.2)	48.7 (6.7)	49.8 (6.3)	<0.001
Smoking metrics						
Age at smoking debut, years		20.0 (2.3)	20.8 (2.7)	20.5 (2.9)	19.9 (2.1)	0.253
Pack-years of smoking^c^		21.5 (20.1–23.0)	13.0 (12.0–14.0)	27.8 (27.0–28.5)	50.0 (48.3–51.8)	<0.001
Median years since quitting		11				
Physically active^a^, %	52.4	64.2	55.3	55.1	54.6	0.314
Alcohol consumption, g/day^c^	4.2 (3.8–4.6)	10.0 (9.0–11.2)	8.8 (7.1–11.1)	13.1 (11.3–15.4)	16.1 (13.4–19.5)	<0.001
Body mass index, kg/m^2^	22.5 (2.7)	23.2 (2.5)	23.1 (2.8)	23.0 (2.7)	23.1 (2.6)	<0.001
Sleep duration, minutes/day	387.0 (51.5)	402.4 (49.2)	398.3 (49.9)	400.1 (55.5)	395.9 (53.8)	<0.001
Dietary factors						
Fat consumption, g/day	56.9 (20.0)	57.4 (20.2)	55.7 (22.5)	54.2 (20.1)	52.4 (21.3)	<0.001
Energy, kcal	1891.9 (579.6)	2015.5 (583.6)	1966.5 (617.8)	2001.6 (587.7)	1953.8 (610.7)	0.001
Carbohydrate consumption, g/day	254.9 (87.1)	264.3 (91.0)	259.1 (85.8)	261.5 (85.2)	256.0 (94.2)	0.344
Sugar consumption, mg/day^c^	11.6 (11.2–12.0)	10.2 (9.6–10.8)	9.4 (8.5–10.4)	10.2 (9.5–11.0)	9.6 (8.6–10.7)	<0.001
Glycemic load	154.5 (59.1)	158.6 (62.1)	156.6 (58.2)	156.9 (59.3)	153.9 (65.3)	0.704

Each continuous variable is being reported as mean (SD) unless mentioned otherwise.

^a^Physical activity: self-reported exercise for a total of 60 minutes or more for more than a day per month.

^b^Using ANOVA and Chi-square test for continuous and categorical variables, respectively.

^c^Values are expressed as geometric mean (95% confidence interval).

**Table 2.** Subjects’ baseline levels of metabolic risk factors, serum adiponectin, leptin, and CRP by smoking status, Aichi, 2002–2011

Characteristics	Smoking status at recruitment					*P*-value^a^

	Never smokers	Ex-smokers	Current smokers, by cigarettes per day			

			Light smokers (<20)	Moderate smokers (20–<30)	Heavy smokers (30+)	
*n*	1608	776	264	421	269	
Positive family history of DM^b^, %	16.5	13.3	12.9	14.5	14.9	0.192
Total cholesterol, mg/dL	211.5 (33.7)	212.9 (33.5)	210.1 (37.3)	208.5 (34.3)	205.7 (33.4)	0.006
HDL cholesterol, mg/dL	64.6 (15.3)	61.1 (15.4)	59.2 (14.7)	56.5 (15.3)	53.4 (13.7)	<0.001
Triglycerides, mg/dL^e^	88.7 (86.4–91.1)	107.2 (103.2–111.5)	110.7 (103.0–118.9)	121.5 (115.3–128.0)	131.6 (122.8–141.0)	<0.001
Total-to-HDL cholesterol ratio	3.4 (0.98)	3.7 (1.00)	3.8 (1.1)	3.9 (1.1)	4.1 (1.2)	<0.001
Fasting blood sugar, mg/dL	89.2 (10.5)	92.1 (10.6)	90.5 (10.7)	91.3 (11.2)	90.7 (11.6)	0.001
Fasting plasma insulin, µU/mL^e^	6.1 (6.0–6.3)	6.4 (6.1–6.7)	6.3 (5.8–6.9)	6.3 (5.9–6.7)	6.1 (6.0–6.7)	0.632
Insulin resistance, HOMA2-IR^e^	0.68 (0.66–0.70)	0.72 (0.68–0.75)	0.70 (0.64–0.77)	0.70 (0.65–0.75)	0.68 (0.62–0.75)	0.565
Hypertensive^c^, %	15.5	26.5	14.0	18.5	24.5	0.006
Systolic blood pressure, mm Hg	123.8 (14.6)	129.1 (15.1)	124.5 (14.6)	126.3 (15.2)	128.3 (16.4)	<0.001
Diastolic blood pressure, mm Hg	75.6 (11.3)	79.9 (11.7)	76.4 (10.5)	78.3 (10.8)	79.2 (12.2)	<0.001
Mean arterial pressure^d^, mm Hg	91.7 (11.7)	96.3 (12.1)	92.4 (11.0)	94.3 (11.5)	95.6 (13.0)	<0.001
Adiponectin, µg/mL^e^	7.7 (7.5–7.8)	6.3 (6.1–6.5)	6.3 (5.9–6.7)	5.9 (5.6–6.1)	5.7 (5.4–6.0)	<0.001
CRP, mg/dL^e^	0.031 (0.029–0.033)	0.036 (0.034–0.039)	0.034 (0.030–0.040)	0.043 (0.039–0.048)	0.064 (0.056–0.073)	<0.001
Leptin, ng/mL^e^	5.3 (5.1–5.4)	4.3 (4.2–4.5)	4.5 (4.2–4.8)	3.8 (3.6–4.0)	3.8 (3.5–4.0)	<0.001
Incident diabetes by 2011, %	4.7	8.4	6.4	8.6	11.9	<0.001

Each continuous variable is being reported as mean (standard deviation) unless mentioned otherwise.

^a^Using ANOVA and Chi-square test for continuous and categorical variables, respectively.

DM, diabetes mellitus; HDL, high-density lipoprotein; HOMA2-IR, homeostatic model assessment 2–insulin resistance; CRP, C-reactive protein.

^b^History of diabetes or raised blood glucose in one or more of first-degree relatives.

^c^Hypertension is defined as systolic/diastolic blood pressure of ≥140/90 mm Hg and/or use of antihypertensive medication.

^d^Diastolic blood pressure + 1/3 (systolic blood pressure-diastolic blood pressure).

^e^Values are expressed as geometric mean (95% confidence intervals).

### Smoking status and risk of diabetes

Compared with the hazard ratio for subjects who never smoked, the age- and sex-adjusted hazard ratios of DM analyzed in aggregate were 1.62 (95% CI 1.13–2.34) among ex-smokers and 1.82 (95% CI 1.29–2.56) among current smokers. After further adjustment for physical activity, family history of DM, mean arterial pressure, and BMI, as well as log-transformed levels of HOMA2-IR, triglycerides, and adiponectin, the hazard ratios for DM were slightly attenuated but remained significantly elevated among both ex-smokers (HR 1.54, 95% CI 1.07–2.22) and current smokers (HR 1.75, 95% CI 1.25–2.46).

Analysis for current smokers stratified by the number of cigarettes smoked per day revealed a dose-dependently increased risk of developing the disease: compared with never smokers, the hazard ratios of DM were 1.35 (95% CI 0.79–2.32) among light smokers, 1.68 (95% CI 1.10–2.58) for moderate smokers, and 2.30 (95% CI 1.47–3.60) for heavy smokers in the maximally adjusted model (Table 3). Similar results of association between smoking status and DM incidence were produced when the analysis was done in a logistic model as part of the mediation analysis (*c* paths in Table 4).

**Table 3.** Hazards of diabetes mellitus by baseline smoking status, Aichi, 2002–2011

	Smoking status at recruitment					

	Never smokers	Ex-smokers	Current smokers, by cigarettes per day			

			Light smokers	Moderate smokers	Heavy smokers	All current smokers
*n*	1608	776	264	421	269	954
Number of cases	75	65	17	36	32	85
Person-years	12 264	5855	1977	3134	1910	7021
Crude incidence rate/1000 person-years	6.1	11.1	8.6	11.5	16.8	12.1
Model 1^a^	1	1.62 (1.13–2.34)	1.36 (0.80–2.33)	1.76 (1.15–2.69)	2.45 (1.56–3.82)	1.82 (1.29–2.56)
Model 2^b^	1	1.64 (1.14–2.36)	1.35 (0.79–2.32)	1.74 (1.13–2.66)	2.41 (1.54–3.77)	1.80 (1.28–2.53)
Model 3^c^	1	1.54 (1.07–2.23)	1.39 (0.82–2.38)	1.74 (1.14–2.67)	2.44 (1.57–3.82)	1.83 (1.30–2.57)
Model 4^d^	1	1.54 (1.07–2.22)	1.35 (0.79–2.32)	1.68 (1.10–2.58)	2.30 (1.47–3.60)	1.75 (1.25–2.46)

^a^Adjusted for age (continuous) and sex at baseline.

^b^Model 1 + adjustments for physical activity, consumptions of alcohol, sugar and energy (all continuous), and sleep duration/day (continuous).

^c^Model 2 + adjustments for family history of diabetes mellitus, mean arterial pressure, body mass index (continuous), total cholesterol to high-density lipoprotein cholesterol ratio, and log-transformed values of homeostatic model assessment 2–insulin resistance (HOMA2-IR) and triglyceride.

^d^Model 3 + adjustment for log-transformed serum adiponectin, C-reactive protain, and leptin levels (continuous).

**Table 4.** Associations among smoking status, diabetes, and three potential mediators in a multiple mediation model, Aichi, 2002–2011

Antecedent	Consequent											

	Adiponectin (M_1_)			Leptin (M_2_)			CRP (M_3_)			DM incidence (Y)		
Smoking status (X)	Path	β (SE)	*P*-value	Path	β (SE)	*P*-value	Path	β (SE)	*P*-value	Path	β (SE)	*P*-value

Never smokers	Referent											
Ex-smokers	*a_11_*	−0.011 (0.009)	0.194	*a_21_*	−0.002 (0.008)	0.793	*a_31_*	−0.019 (0.022)	0.392	*c_1_*	0.485 (0.196)	0.014
Light smokers	*a_12_*	−0.033 (0.014)	0.017	*a_22_*	−0.006 (0.011)	0.564	*a_32_*	−0.013 (0.032)	0.68	*c_2_*	0.330 (0.286)	0.249
Moderate smokers	*a_13_*	−0.044 (0.011)	<0.001	*a_23_*	−0.035 (0.01)	<0.001	*a_33_*	0.076 (0.027)	0.005	*c_3_*	0.605 (0.229)	0.008
Heavy smokers	*a_14_*	−0.054 (0.013)	<0.001	*a_24_*	−0.034 (0.012)	0.003	*a_34_*	0.235 (0.032)	<0.001	*c_4_*	0.927 (0.243)	<0.001
Adiponectin (M_1_)	*b_1_*										−0.992 (0.381)	0.009
Leptin (M_2_)	*b_2_*										0.134 (0.445)	0.763
CRP (M_3_)	*b_3_*										−0.062 (0.158)	0.693

CRP, C-reactive protein; DM, diabetes mellitus; SE, standard error.

### Association between smoking and adiponectin, leptin, and CRP

Table 4 shows the association of each smoking category, relative to never smokers, with concentrations of adiponectin, leptin, and CRP. Significant inverse associations between current smoking and adiponectin levels were observed. The magnitudes of association with being a light, moderate, or heavy smoker were (β [SE] = −0.03 [0.01], *P* = 0.017), (β [SE] = −0.04 [0.01], *P* < 0.001) and (β [SE] = −0.05 [0.01], *P* < 0.001), respectively. Adiponectin concentrations in ex-smokers, however, were not significantly different from those in never smokers (β [SE] = −0.01 [0.01], *P* = 0.194). Leptin concentrations were negatively associated with all smoking categories, although the associations were significant only among moderate (β [SE] = −0.035 [0.01], *P* < 0.001) and heavy smokers (β [SE] = −0.034 [0.012], *P* = 0.003). Concentrations of CRP were negatively but non-significantly associated with ex- and light smokers. In contrast, CRP concentration was positively and significantly associated with being a moderate (β [SE] = 0.076 [0.027], *P* = 0.005) or heavy smoker (β [SE] = 0.235 [0.032], *P* < 0.001).

### Association between DM incidence and adiponectin, leptin, and CRP

Adiponectin concentration was inversely and significantly associated with DM incidence (β [SE] = −0.992 [0.381], *P* = 0.009). However, concentrations of leptin (β [SE] = 0.134 [0.445], *P* = 0.763) and CRP (β [SE] = −0.062 [0.158], *P* = 0.693) were not significantly associated (Table 4). These associations with adiponectin, leptin, and CRP concentrations were independent of one another and all other covariates described above.

### Mediating role of adiponectin, leptin, and/or CRP

Table 5 shows the direct effects, and the indirect effects through the three potential mediators, of being in each smoking category on DM incidence relative to never smokers. When adjustments for the three potential mediators were made simultaneously, the direct effects of being an ex-, moderate, and heavy smoker on DM incidence remained significant with β (SE) = 0.480 (0.196), *P* = 0.015; β (SE) = 0.574 (0.230), *P* = 0.013; and β (SE) = 0.880 (0.247), *P* < 0.001, respectively.

**Table 5.** Direct and indirect effects though three potential mediators of smoking status on diabetes incidence, Aichi, 2002–2011

Smoking status	Direct effect of Smoking on DM incidence (*c′* paths)			Indirect effect of smoking on DM incidence (path *ab*’s)					
	
	Path	β (SE)	95% CI	Through Adiponectin (M_1_)		Through Leptin (M_2_)		Through CRP (M_3_)	
		
				Point estimate (SE)	BC 95% CI^a^	Point estimate (SE)	BC 95% CI^a^	Point estimate (SE)	BC 95% CI^a^
Never smoker	Referent								
Ex-smoker	*c′_1_*	0.480 (0.196)	0.095 to 0.864	0.011 (0.010)	−0.003 to 0.040	−0.0003 (0.004)	−0.012 to 0.006	0.001 (0.004)	−0.004 to 0.017
Light smokers	*c′_2_*	0.299 (0.286)	−0.263 to 0.860	0.033 (0.019)	0.005 to 0.082	−0.0009 (0.006)	−0.020 to 0.007	0.0008 (0.005)	−0.006 to 0.020
Moderate smokers	*c′_3_*	0.574 (0.230)	0.122 to 1.025	0.044 (0.021)	0.010 to 0.094	−0.0047 (0.017)	−0.041 to 0.027	−0.0047 (0.012)	−0.033 to 0.015
Heavy smokers	*c′_4_*	0.880 (0.247)	0.396 to 1.365	0.054 (0.025)	0.013 to 0.113	−0.005 (0.017)	−0.042 to 0.027	−0.015 (0.034)	−0.086 to 0.049

BC, bias-corrected; CI, confidence interval; CRP, C-reactive protein; SE, standard error.

^a^Bias-corrected confidence intervals after running 10 000 bootstrap samples.

Adiponectin levels appeared to partially mediate the association between the three categories of current smoking and DM incidence: the indirect effects of being a light (point estimate 0.033; BC 95% CI 0.005–0.082), moderate (point estimate 0.044; BC 95% CI 0.010–0.094) or heavy smoker (point estimate 0.054; BC 95% CI 0.013–0.113) on DM, relative to never smokers, were statistically significant. In terms of the ratio of indirect effect to total effect, the contribution of adiponectin concentration as a mediator in the smoking-DM association was 10%, 7.2%, and 5.8% among light, moderate, and heavy smokers, respectively. In contrast, neither levels of leptin nor CRP seemed to mediate the smoking-DM association, as the corresponding BC 95% CIs included zero (Table 5).

### Additional analyses

We also conducted mediation analyses using other cardiovascular risk factors, including lipids, glucose, and blood pressure, which were significantly associated with smoking in our study. However, the indirect effects of smoking on DM incidence were statistically insignificant when each of those variables was used as a potential mediator in the model (data not shown).

## DISCUSSION

The main findings of our study are threefold. First, we confirmed that risk of developing DM was significantly elevated among ex- and current smokers. Second, we found that adiponectin levels were inversely associated with both smoking and DM incidence, and that leptin and CRP levels were associated with smoking but not with DM. Finally, of the three potential mediators assessed, we found that only adiponectin mediated the association between current smoking and DM.

A positive association between active smoking and DM incidence has been noted in a number of studies conducted among various populations,^44^^–^^52^ as well as in a recent meta-analysis of 25 prospective cohort studies.^1^ The substantial increase in the risk of DM among current smokers that we observed in our study is consistent with these previous reports. Our results affirm the putative influence of active smoking on the incidence of DM. Being an ex-smoker was also significantly associated with increased risk of DM compared to never smokers in the present study, although the risk is far less than the one exhibited among current heavy smokers. An increased risk of DM among ex-smokers’ has been shown in some^44^^,^^53^^,^^54^ but not all^47^^,^^50^^,^^52^^,^^55^ previous studies. The discrepancy might be related to differences in ex-smokers’ intensity of smoking and intervals since quitting among subjects in the respective studies.

Serum adiponectin levels were inversely associated with both current smoking and DM incidence in our study. This corroborates the findings of previous studies that reported the inverse associations of adiponectin levels independently with active smoking^6^^–^^10^ and DM incidence.^11^^–^^14^ The present study revealed, for the first time, that adiponectin may play a significant mediating role in the smoking-DM association; the effect of being a light, moderate, or heavy smoker on DM incidence may be due in part to its indirect effect through adiponectin. However, the indirect effect of being an ex-smoker on DM through adiponectin was not significant. These results may indicate that the effect of smoking on adiponectin is short-lived after smoking cessation, as shown in previous studies that found that ex-smokers’ serum adiponectin were able to increase^56^^,^^57^ and be restored to normal levels in as little as 2 months after quitting smoking.^57^

There are several biologically plausible mechanisms that may explain how the speculated smoking-adiponectin-DM causal pathway might work. Smoking-induced oxidative stress decreases the secretion and expression of plasma adiponectin via inhibition of the activation of phosphatidylinositol 3-kinase,^9^ a key molecule in the secretion of adiponectin in adipocytes.^58^ Nicotine in tobacco smoke can also directly inhibit the secretion and expression of adiponectin in adipocytes.^9^^,^^59^ Moreover, persistent production of tumor necrosis factor α induced by chronic exposure to cigarette smoke may promote the development of hypoadiponectinemia.^60^^,^^61^ Hypoadiponectinemia may, in turn, cause insulin resistance and DM; adiponectin is believed to stimulate the phosphorylation and activation of 5′-adenosine monophosphate-activated protein kinase in the liver and skeletal muscles, thereby directly regulating glucose metabolism and insulin sensitivity.^4^^,^^62^ Adiponectin may also increase fatty-acid combustion and energy consumption, in part via peroxisome proliferator-activated receptor α activation, leading to decreased triglyceride content and a corresponding coordinated increase in insulin sensitivity in the liver and skeletal muscles.^4^^,^^63^

Contrary to our initial hypothesis, neither leptin nor CRP appeared to mediate the smoking-DM association. This finding ruled out the smoking-leptin-DM or smoking-CRP-DM pathways as alternative causal pathways in the smoking-DM association. There are hardly any previous studies on this issue with which to compare our findings. However, the present finding that only adiponectin partially mediated the smoking-DM association may indicate that smoking is related to DM development through adipocyte dysfunction^64^ secondary to smoking-induced adipocyte inflammation, and leptin and CRP are likely markers of such a state.

Moderate and heavy smokers had significantly lower leptin but higher CRP levels relative to never smokers in our study. Although previous studies on the influence of smoking on leptin are few, findings in available reports were inconsistent: increasing,^20^^,^^21^ reducing,^15^^–^^18^ or no effects^19^ of smoking on leptin were documented. One of the reasons for such a discrepancy might be the fact that smoking may interact with other factors such as diet, exercise, and other lifestyle factors, as well as hormones and host inflammatory responses, and these interactions may further impair leptin regulations.^15^^,^^16^ Further studies are warranted to elucidate how these interactions may mediate the effect of smoking on leptin levels. With regard to the smoking-CRP association, the significantly elevated levels of CRP observed among moderate and heavy smokers in our study was consistent with previous findings,^29^^–^^33^ reinforcing the acknowledged pro-inflammatory impact of smoking.^65^

Neither leptin nor CRP levels were significantly associated with DM incidence in the present study. Findings of previous studies investigating the association of both biomarkers individually with DM have been equivocal: independent positive associations with the disease of either leptin or CRP were reported in some studies^22^^–^^24^^,^^34^^–^^36^^,^^66^ but not all.^25^^–^^27^^,^^37^^,^^38^ These inconsistencies may be due in part to variations in the potential confounders considered among studies, in addition to differences in population characteristics. For instance, the lack of a significant association with DM incidence of both leptin and CRP observed in our study was independent of adiponectin; this was not the case in previous studies reporting a positive association.^22^^–^^24^^,^^34^^,^^36^^,^^66^ Conversely, the results of studies that reported no significant association between DM and leptin or CRP levels were obtained after controlling for the possible effect of adiponectin.^27^^,^^38^ Since adiponectin is associated with both leptin and CRP,^67^^,^^68^ we speculate that adiponectin might negatively confound the association of leptin with DM and qualitatively confound the association of CRP with DM. Further studies specifically designed to test this hypothesis are warranted.

Our study has several limitations. First, there may have been misclassification bias, as smoking status was self-reported. However, we believe that any such bias would be non-differential and not expected to affect the results significantly. Second, some of the participants in the cohort were censored at the time of retirement. However, we believe censoring due to retirement was non-differential to the outcome and would not introduce significant bias. Third, the analysis of the association of smoking status with the three biomarkers was cross-sectional, and claims of causality should be made with caution. But the possibility of reverse causality is remote, as the levels of the biomarkers are not likely to influence smoking behavior. Fourth, total serum adiponectin was used in our analysis. The high-molecular-weight complex of adiponectin is believed to correlate with glucose tolerance similarly to or better than total serum adiponectin.^69^^,^^70^ Finally, our study was conducted in middle-aged Japanese workers; hence, further research is needed before the findings are extrapolated to other groups of subjects and races.

However, despite those limitations, our findings have the following clinical and public health implications. First, they contribute to the understanding of the pathogenesis of smoking-related DM and could aid clinicians and public health workers in advising smoking clients. Second, they indicate that an adiponectin-focused intervention in the future may help avert at least 6% of smoking-related cases of DM among current smokers. The public health implications of such an intervention could be even bigger in populations with high prevalence of smoking. In fact, the potential therapeutic role of adiponectin in the treatment of DM, insulin resistance, metabolic syndrome, and cardiovascular diseases has long been documented.^71^^,^^72^ It might also be useful to explore other lifestyle behaviors and traits of individuals that decrease or increase adiponectin concentrations and investigate them in relation to DM incidence. Such studies may identify other targets of DM prevention.

In conclusion, in addition to confirming the positive association between smoking and DM incidence, our study has revealed that serum levels of adiponectin may mediate the association, at least in part. However, levels of leptin and CRP did not appear to have a mediating role in the smoking-DM association nor were they associated individually with DM incidence. Further research is needed to elucidate the role of these biomarkers and their interaction in the pathogenesis of smoking-related DM.

## ONLINE ONLY MATERIAL

Abstract in Japanese.
