# Supplementary material for: Smoking and Diabetes: Is the Association Mediated by Adiponectin, Leptin, or C-reactive Protein?
Source: J Epidemiol. 2015 Feb 5;25(2):99–109. doi: 10.2188/jea.JE20140055 (PMC4310870; doi:10.2188/jea.JE20140055)
Supplement: Abstract in Japanese. [file je-25-099-s001.pdf]

# 喫煙による糖尿病リスクの上昇はアディポネクチン、レプチン、C 反応性タンパクによって仲介されるか？

ヒラウイ H. イサヤス [1,2], 八谷寛 [1,3], 李媛英 [3], 上村真由 [1], 王超辰 [1], 江啓発 [1], 豊嶋英明 [4], 玉腰浩司 [5], 張燕 [1], 川副延生 [1], 青山温子 [1]
1 名古屋大学大学院医学系研究科国際保健医療学・公衆衛生学
2 エチオピア メケレ大学　公衆衛生学
3 藤田保健衛生大学医学部公衆衛生学
4 安城更生病院
5 名古屋大学大学院医学系研究科看護学専攻

背景：喫煙が 2 型糖尿病発症リスクに関連することはよく知られているが、そのメカニズムは十分解明されていない。そこで、その関連がアディポネクチン、レプチン、C 反応タンパク（CRP）によって説明されるか検討した。
方法：愛知職域コホート（第 2 次）に参加した 35-66 歳の男性従業者 3,338 名を 2002 年から 2011 年まで追跡した。多変量調整 Cox 回帰モデルを用いてベースライン時喫煙状況の糖尿病発症に対するハザード比と 95%信頼区間を計算した。喫煙が上記 3 つのバイオマーカーを介して糖尿病発症リスクに与える間接的な「影響」の大きさは多変量仲介モデルを用いて推定し、そのバイアス調整 95%信頼区間（BC 95%CI）はブートストラップ法によって求めた。
結果：非喫煙者に比し、現喫煙者の糖尿病発症リスクは有意に高かった（ハザード比：1.75、95%CI：1.25-2.46）。喫煙の糖尿病発症に対するアディポネクチンを介する「影響」の大きさの推定値（BC 95%CI）は、軽度、中等度、重度の喫煙でそれぞれ 0.033（0.005-0.082）、0.044（0.010-0.094）、0.054（0.013-0.113）であった。レプチンや CRP を介する影響は統計学的に有意ではなかった。
結論：喫煙の糖尿病発症に対する「影響」の一部はアディポネクチンによって仲介されていると考えられた。

キーワード: アディポネクチン; C 反応タンパク; レプチン; 仲介解析; 喫煙, 2 型糖尿病
